# Orthostatic Stress and Baroreflex Sensitivity: A Window into Autonomic Dysfunction in Lone Paroxysmal Atrial Fibrillation

**DOI:** 10.3390/jcm12185857

**Published:** 2023-09-08

**Authors:** Mónica Ferreira, Sérgio Laranjo, Pedro Cunha, Vera Geraldes, Mário Oliveira, Isabel Rocha

**Affiliations:** 1 Faculdade de Medicina and Centro Cardiovascular da Universidade de Lisboa—CCUL, Universidade de Lisboa, 1649-004 Lisbon, Portugal; ferreira_t_monica@hotmail.com (M.F.); vgeraldes@medicina.ulisboa.pt (V.G.); 2 Arrhythmology, Pacing and Electrophysiology Unit, Serviço de Cardiologia, Hospital de Santa Marta, Centro Hospitalar Universitário de Lisboa Central—CHULC, 1150-199 Lisbon, Portugal; sergio.laranjo@chlc.min-saude.pt (S.L.); pedro.cunha@chlc.min-saude.pt (P.C.); marioj.oliveira@chlc.min-saude.pt (M.O.); 3 CHRC, NOVA Medical School, Faculdade de Ciências Médicas, NMS, FCM, Universidade NOVA de Lisboa, 1169-056 Lisboa, Portugal

**Keywords:** atrial fibrillation, baroreceptor reflex, autonomic function, baroreflex gain, baroreflex effectiveness index

## Abstract

The abnormal neural control of atria has been considered one of the mechanisms of paroxysmal atrial fibrillation (PAF) pathogenesis. The baroreceptor reflex has an important role in cardiovascular regulation and may serve as an index of autonomic function. This study aimed to analyze the baroreceptor reflex’s role in heart rate regulation during upright tilt (HUT) in patients with lone PAF. The study included 68 patients with lone PAF and 34 healthy individuals who underwent baroreflex assessment. Parameters such as baroreflex sensitivity (BRS), number of systolic blood pressure (BP) ramps, and the baroreflex effectiveness index (BEI) were evaluated. The study found that PAF patients had comparable resting BPs and heart rates (HRs) to healthy individuals. However, unlike healthy individuals, PAF patients showed a sustained increase in BP with an upright posture followed by the delayed activation of the baroreceptor function with a blunted HR response and lower BEI values. This indicates a pronounced baroreflex impairment in PAF patients, even at rest. Our data suggest that together with BRS, BEI could be used as a marker of autonomic dysfunction in PAF patients, making it important to further investigate its relationship with AF recurrence after ablation and its involvement in cardiovascular autonomic remodeling.

## 1. Introduction

Atrial fibrillation (AF), a highly prevalent type of cardiac arrhythmia in clinical settings, considerably affects health outcomes. It often correlates with detrimental effects on life span and quality of life, posing a considerable public health concern [1,2,3,4,5,6]. Around 30% of all AF instances manifest as paroxysmal AF (PAF), characterized as self-ending episodes of the arrhythmia lasting less than 7 days [7,8,9,10]. 

Understanding the intricate physiopathology of AF remains a complex and challenging task. One reason is the multitude of mechanisms underpinning its initiation and maintenance. For example, lone AF is associated with a sequence of electrophysiological events that involve the participation of numerous wavelets, which spread and randomly interact across the surface of the atrium. Furthermore, these wavelets may be influenced by focal sources of electrical activity referred to as “triggers”. It has been proposed that these triggers could be affected by combined sympathovagal discharges, further complicating this scenario [11,12,13].

The autonomic nervous system (ANS) plays a central role in the pathogenesis of AF and influences atrial electrophysiology and function [14,15,16,17]. Both vagal and sympathetic activations can alter the electrophysiological properties of the atrium, leading to AF episodes [18,19,20]. This interaction between the two arms of the ANS is often referred to as autonomic remodeling.

The baroreceptor reflex, which is crucial for short-term blood pressure regulation, serves as an autonomic index of function [21]. While some studies suggest decreased baroreflex sensitivity (BRS) in AF patients, suggesting altered ANS cardiac control [21,22], the relationship between PAF and baroreflex modulation remains unclear. In particular, BRS changes after AF ablation procedures could predict the success of these procedures [23,24,25,26,27].

In light of these gaps in understanding, our study aimed to explore the behavior of baroreceptor reflex control in patients with lone PAF, both at rest and during an upright tilt (HUT) through the evaluation of BRS and BEI. This approach could provide valuable insights into the complex interplay between autonomic regulation and atrial fibrillation, paving the way for more targeted therapeutic strategies.

## 2. Materials and Methods

### 2.1. Subjects 

In this prospective research study, which focuses on elucidating the role of the baroreceptor reflex in cardiovascular regulation during an HUT in patients diagnosed with lone paroxysmal atrial fibrillation (PAF), and which was conducted at the Arrhythmology Department of Santa Marta Hospital in Lisbon, Portugal, we recruited adult patients of both sexes who had suffered from lone PAF for at least one year. A careful history was taken for each patient, followed by confirmatory tests with electrocardiograms (ECGs) and Holter recordings. All patients were characterized by lone PAF, without clinical or echocardiographic signs of concomitant cardiovascular disease. This strict inclusion criterion was set to exclude potential confounding variables and to ensure the accuracy of the study. Exclusion criteria were clearly defined so that all patients with a history of syncope, myocardial infarction, congestive heart failure, renal failure, diabetes mellitus, hypertension, and sleep apnea were excluded. In addition, patients under the effect of beta-blockers or those who had experienced an episode of AF within the 48 h before the study were excluded. Antiarrhythmic drugs, if present, were discontinued for at least five half-lives before the baroreflex sensitivity (BRS) and effectiveness (BEI) assessment were performed. In addition, the study also included a control group of healthy adults of both sexes who were closely matched for age, gender, and other relevant demographic factors. This meticulous selection process was aimed at ensuring a balanced and rigorous comparison between the two cohorts. 

Following data protection regulations and policies, all patient data used in this study were collected, stored and analyzed securely and confidentially. Patient data were anonymized and de-identified before analysis to protect privacy. The research report does not contain any identifiable personal information. Ethical approval was obtained from the ethics committee of Centro Hospitalar Universitario Lisboa Central, and informed consent was obtained from all participants. The study strictly adhered to the principles of the Declaration of Helsinki and its subsequent amendments, as well as Portuguese data protection laws and regulations (Internal IRB Ref 1158/13).

### 2.2. Tilt Test 

The participants were instructed not to consume alcohol or tobacco and not to take any medication with anticholinergic properties, at least in the 12 h before the test. HUT was performed in a dedicated lab, in a quiet environment, with controlled temperature and humidity, during the morning period, following four hours of fasting. Briefly, the subjects were placed on a tilt table with a footboard, secured with snug restraints to prevent falling if syncope occurred, and, after a resting period of 15 min in the supine position (0°, were tilted head-up using an electronically operating system to a level of 70° at a constant speed (within 15 s). After the end of the 5th minute in the orthosthatic position, the subjects were tilted down. All subjects were instructed to breathe normally during the full length of the test. ECG and blood pressure (BP) were continuously monitored using a Task Force Monitor (CNSystems, Graz, Austria).

### 2.3. Data Analysis

All signals were acquired using the Taskforce Monitor (CNSystems, Graz, Austria). The ECG was sampled at 1000 Hz with an accuracy of +/−5 V, synchronized with continuous blood pressure measurements sampled at 100 Hz. The raw data underwent preprocessing to minimize interference. Power line interference was attenuated using a 3rd-order notch filter (49–51 Hz). Baseline wander, typically <0.5 Hz, was reduced using a high-pass second-order Butterworth filter. Muscle noise was addressed using a low-pass filter with a 35 Hz cut-off. A wavelet-based QRS detector followed by an adaptive dual-threshold algorithm was employed for waveform detection. After QRS detection using a Daubechies 12 discrete wavelet transform, the adaptive double threshold algorithm was applied. This ensured robust peak detection and accurate results with a maximum error tolerance of 1–2 ms. A waveform typing algorithm differentiated between QRS complex morphologies, identifying the dominant morphology and distinguishing it from other beats. Ectopic beats or outliers were replaced using cubic spline interpolation.

Continuous RR intervals (RRIs) and non-invasive BPs were obtained, and data analysis was performed on three periods: (1) the last 4 min of the supine resting period (0, baseline); (2) during the first minute of tilt adaptation at 70° (TA1); and (3) in the fifth minute after tilting (TA5). 

#### Arterial Baroreflex Function

Systolic BPs (sBPs) and RRIs were used for the subsequent analysis of baroreflex function through the sequence method (BRS) as stated elsewhere [28,29,30]. Briefly, BRS estimation was based on the analysis of beat-to-beat series of sBPs, scanned in order to identify ramps of three or more consecutive heart beats with a progressive increase or decrease of at least 1 mmHg, regardless of the possible occurrence of concomitant RRI changes. The algorithm identified sequences, defined as sBP ramps followed by concordant pulse interval variations of 5 ms, coupled with 0-, 1-, and 2-beat lags, with each sequence being included only once [29,30]. For each sequence, the average regression slope of the linear inter-relationship between sBP and the following RRI values was calculated and considered reliable when the correlation coefficient was greater than 0.80. The mean slope of all regression lines in a stationary phase was used as a marker of BRS.

For each period considered for analysis, BEI, which reflects the number of times the arterial baroreflex is in control of the heart rate response to blood pressure fluctuations and is defined as the ratio between the number of BRS sequences detected and the total number of sBP ramps observed, was calculated [28].

### 2.4. Statistical Analysis

Continuous variables are expressed as mean ± standard error of the mean (SEM), unless otherwise specified. Categorical variables are given as frequencies and percentages, and the chi-square test was used to determine if there was any association between variables. The normality of the distributions of the continuous variables was analyzed with the Kolmogorov–Smirnov test. A two-way ANOVA analysis of variance with repeated measures was used to compare data along the test and between groups. A value of *p* < 0.05 was considered statistically significant. To rigorously assess the potential impact of confounding variables on our primary outcomes of BRS and BEI, a multiple regression analysis was employed to control for age, BMI, baseline BP, and HR as covariates. Each variable was included in the regression model to evaluate its independent effect on the outcomes while adjusting for the other variables. The significance of each variable was tested, and the adjusted effect sizes (beta coefficients) were computed. In addition, a sensitivity analysis was computed to examine the finding’s robustness. For that, each potential confounding variable was sequentially excluded from the regression model, and the resulting effect sizes and *p*-values were compared to the original model. This sensitivity analysis was carried out for both BRS and BEI to ensure that our findings were not unduly influenced by any single confounding variable. Data was analyzed using GraphPAD Instruments version 3.05 (GraphPad Software, Inc., La Jolla, CA, USA).

## 3. Results 

### 3.1. Population

The study sample consisted of 68 patients of both sexes, with a mean age of 53 ± 14 years and a documented history of paroxysmal atrial fibrillation (PAF) of at least one year (range 1–5 years), and 34 healthy volunteers matching age and sex served as the control group. All patients were diagnosed with lone PAF, defined as the absence of clinical or echocardiographic evidence of cardiovascular disease. Our study included all participants’ successful completion of the testing protocol, with no recorded symptoms or initiation of AF. Given the matching and exclusion criteria applied, potential confounders were minimized and no further adjustments for confounders were deemed necessary. The evaluation of the baseline characteristics indicated no significant differences between patients with paroxysmal atrial fibrillation (PAF) and healthy individuals (HIs) in terms of age, sex distribution, body mass index, baseline arterial blood pressure (BP), and heart rate (Table 1).

In our study, it was important to consider the possible bias due to the recruitment and selection procedures, which could affect the generalizability of the results to the wider population of patients with lone AF. A detailed analysis of bias was conducted and can be found in the Supplementary Materials, Table S1.

### 3.2. Heart Rate and Blood Pressure Changes

**Figure 1:**
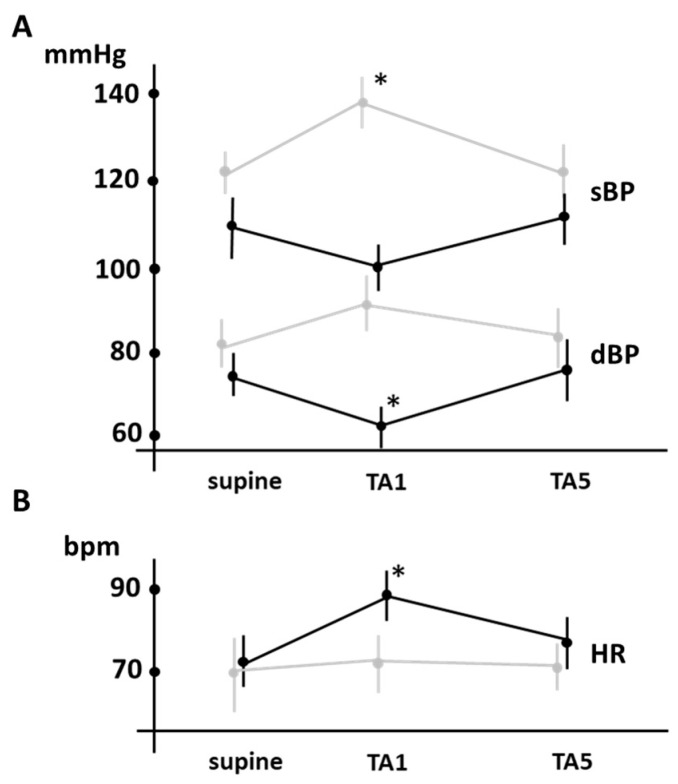
Illustrates the changes in sBP, dBP ((**A**), in mmHg) and HR ((**B**), in bpm) during the tilt test. TA1 and TA5 correspond to the first and fifth minutes of tilt adaptation. A comparative analysis between the groups of PAF patients (light gray) and control subjects (dark gray) revealed no significant differences for HR, but differences were found in sBP and dBP. In particular, a significant difference was found between PAF patients and control individuals in TA1. The asterisk (*) indicates changes that were significant compared to baseline values (*p* < 0.05). These data provide a comprehensive, comparative overview of the physiological responses to the tilt test in PAF patients and control participants.

**Figure 2:**
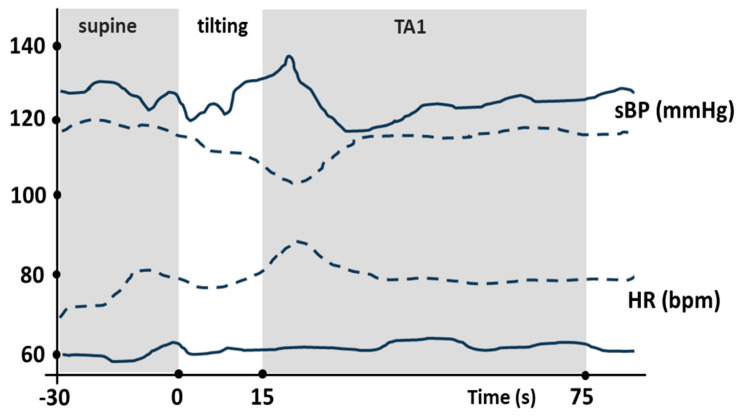
This figure shows the different cardiovascular responses elicited by tilt tests in a 45-year-old healthy control subject (dashed lines) and a 46-year-old PAF patient (solid lines). The upper diagram shows the fluctuations in sBP, while the lower diagram shows the fluctuations in HR. TA1 indicates the first minute of the tilt adjustment. This side-by-side comparison provides a unique insight into the different physiological responses to tilt testing in individuals with and without paroxysmal atrial fibrillations, showing the blunted heart rate response and the delayed pressure adaptation of the PAF patient.

During HUT, the control participants showed an initial, statistically significant drop in BP, which then recovered and subsequently rose again (baseline systolic blood pressure changed from 115 ± 8.3 to 100 ± 5.1 (TA1) (*p* < 0.05) and 116 ± 7.4 (TA5) mmHg, whereas diastolic blood pressure changed from 76 ± 5.5 to 62 ± 4.2 (TA1) and 77 ± 7.3 (TA5) mmHg; n = 34). In contrast, PAF patients experienced a continuous and progressive increase in BP followed by a decrease and a slow adaptation until the recovery of basal values. sBP varied significantly from baseline values of 121 ± 4.3 to 139 ± 5.5 (TA1) (*p* < 0.05) and 122 ± 6.4 (TA5) mmHg, whereas diastolic blood pressure (dBP) changed non-significantly from the baseline values of 81 ± 5.5 to 92 ± 6 (TA1) (*p* < 0.05) and 82 ± 6.2 (TA5) mmHg (n = 68) (see Figure 1). This resulted in significantly increased levels of BP during the first moments of orthostasis in the PAF patients compared to the control subjects (see Figure 1A and Figure 2).

During the transition from baseline to the tilt adaptation periods (TA1 and TA5), heart rate increased in both groups from baseline 70 ± 10.1 to 72 ± 7 (TA1) and to 71 ± 5.7 (TA5) bpm to PAF patients (*n* = 68) and 72 ± 5.8 at baseline to 89 ± 6 (TA1) (*p* < 0.05) and 77 ± 6.7 (TA5) (*n* = 34)). Thus, heart rate responses to HUT were the same in both groups, with no significant difference in the amount of heart rate change between the PAF and control groups throughout the test (Figure 1B and Figure 2).

### 3.3. Baroreflex Sensitivity and Effectiveness

**Figure 3:**
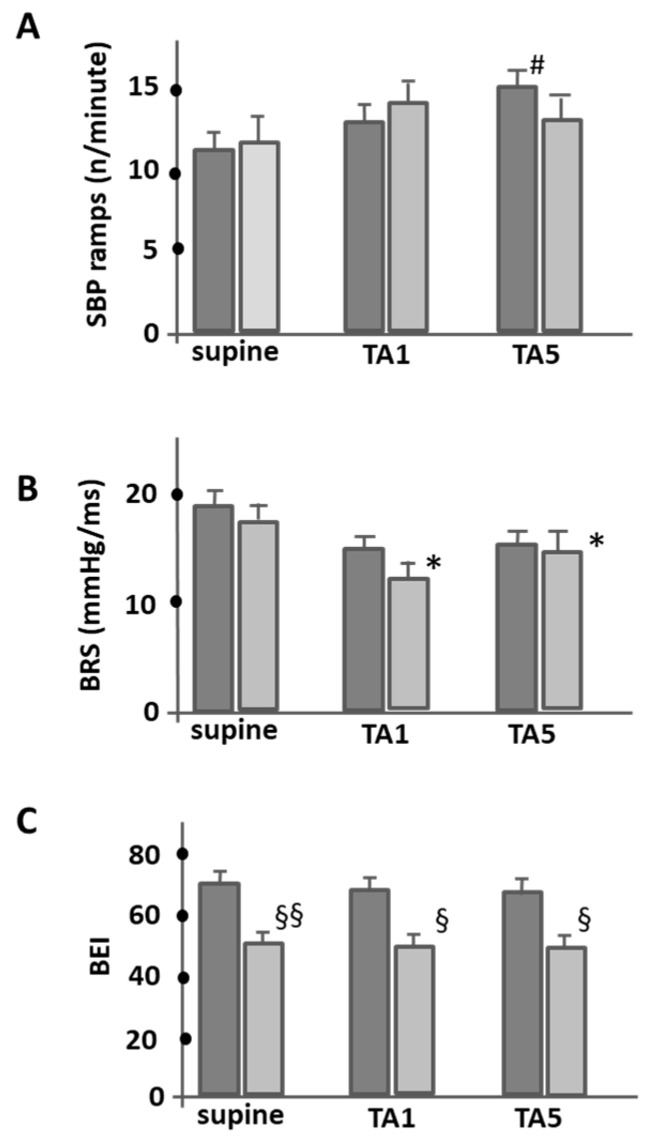
This figure illustrates the dynamic fluctuations in sBP ramps (**A**), BRS (**B**), and BEI (**C**) during the study in PAF patients (light gray) and control subjects (dark gray). These comparative insights allow for a better understanding of baroreflex dynamics in both PAF patients and healthy subjects. # *p* < 0.05 between TA5 and the baseline values for control subjects; * *p* < 0.05 between each group and baseline values for PAF patients; § *p* < 0.01 and §§ *p* < 0.001 for the comparison between groups in the same period.

Figure 3 shows an analysis of the mean ramp frequency of sBP per minute and the corresponding mean BRS and BEI values in the different phases of the HUT protocol, i.e., baseline and the TA1 and TA5 phases. The data provide some fascinating insights. While the analysis of the mean frequency of sBP increases per minute during the different phases of HUT revealed no significant difference between the groups in terms of the number of sBP increases, the control group showed a significant increase in sBP ramp frequency during HUT, especially during the TA5 phase (11.4 ± 1.2 vs. 15 ± 1.3; *p* < 0.05) (Figure 3A). In contrast, the number of ramps in the PAF patients remained significantly unchanged throughout the test.

Spontaneous BRS at baseline was comparable between the control and PAF groups. However, when transitioning to an upright position, both groups showed a decrease in BRS slope, with the decrease being statistically significant in the PAF patients (17.8 ± 1.9 mmHg/ms (supine) vs. 12.1 ± 1.8 (TA1), *p* < 0.05). It is noteworthy that the PAF patients consistently had lower BRS values than the control group during the TA1 and TA5 phases (11.3 ± 1.6 mmHg/ms vs. 15.1 ± 2 mmHg (TA5), *p* < 0.05; Figure 3B). In terms of BEI, the PAF group consistently had significantly lower values than the control group during all test phases, highlighting the potential baroreflex dysfunction in the PAF population (supine; 71 ± 3.8 (PAF) vs. 50 ± 3.7 ms^−1^ (control); *p* < 0.001 and during tilt (at TA1 (68 ± 4 (PAF) vs. 50 ± 3.5 ms^−1^ control, *p* < 0.01 and at TA5 (67 ± 3.5 (PAF) vs. 49 ± 3.7 ms^−1^ (control); *p* < 0.01). 

## 4. Discussion

The baroreceptor reflex stands as one of the most crucial physiological systems facilitating short-term or swift cardiovascular autonomic regulation, gaining particular significance during postural adjustments to mitigate BP fluctuations [21,28,30,31,32]. The primary aim of this study was to assess baroreflex function in PAF patients amidst a passive short HUT, by utilizing the spontaneous sequence method applied to continuous RRI and BP signals. This discussion will break down our findings and elucidate how they contribute to the understanding of baroreflex function in PAF patients during a passive short HUT. The information is, thus, structured around key findings regarding heart rate and blood pressure changes, baroreflex sensitivity, the role of sBP ramps, and the significance of these findings in clinical practice.

### 4.1. Evaluation of Baroreflex Sensitivity in PAF Patients

The spontaneous sequence method applied to continuous RRI and BP signals allows for the evaluation of BP variability, which prompts baroreceptor activation by calculating sBP ramps. It provides information regarding BRS by evaluating the amplitude of the reflex changes in RRIs concerning variations in sBPs [28,32]. In addition, the BEI offers insights into baroreflex function by quantifying the frequency of arterial baroreflex response to progressive sBP changes with concordant RRI modulation changes. 

Our results illustrate significant differences in BRS, BEI, and BP responses in the first and fifth minute of the adaptation period after orthostatism in PAF patients compared to control subjects. Despite comparable clinical characteristics and no differences in the mean HR and BP between groups under baseline conditions, PAF patients exhibited a diminished capacity to regulate BP in response to tilt, as demonstrated by a sustained and progressive increase in sBP in the first minute and a delayed baroreflex activation, which is indicated by a lower BEI than the control subjects, with no remarkable changes in the number of sBP ramps. Additionally, the PAF group demonstrated a more significant reduction in the BRS slope from the first minute of HUT onwards.

Interestingly, the control group demonstrated a significant rise in sBP ramps over the course of the five-minute head-up tilt test. Conversely, patients with PAF patients showed no variations in the count of sBP ramps during the same test. This specific sBP trend might indicate the repeated activation of the arterial baroreflex and could potentially serve as an indicator of BP variability. However, various studies have questioned the relationship between sBP ramps and baroreflex, suggesting that the occurrence of sBP ramps is somewhat unrelated to baroreflex [28,32,33,34]. Therefore, the significance of the sBP ramp dynamic pattern as an indicator of short-term orthostatic adaptation is still unclear. Conflicting results on BRS assessment in patients with PAF have been reported previously [26,27,35,36,37]. Some studies found no difference in BRS between PAF patients and controls [36,37], whereas others have reported reduced spontaneous BRS in symptomatic PAF patients [37]. In addition, our study focuses on comparing PAF patients to healthy individuals, while Miyoshi and colleagues’ study [26] focuses on comparing patients with persistent atrial fibrillation (PeAF) and those with PAF showing that the baseline BRS is significantly lower in PeAF patients compared to PAF patients. Additionally, it reveals that catheter ablation led to a significant decrease in BRS in all patients, but this effect seemed to be less pronounced in patients with PeAF compared to those with PAF. So, while both studies deal with BRS in the context of atrial fibrillation, they focus on different comparisons: one is comparing PAF patients with healthy individuals, while the other is comparing between different types of atrial fibrillation (PAF and PeAF) using, in addition, different methodologies of baroreflex function evaluation. Nevertheless, both studies suggest that AF is associated with the impairment of BRS. Still, the extent and specifics of this impairment seem to vary with the type and stage of atrial fibrillation and with the interventions (like catheter ablation) applied. These variations could be an area of focus for future research in this field. Another interesting study is that of Kondo and colleagues [27], which focused on the impact of pulmonary vein antrum isolation via radiofrequency catheter ablation (RFCA) on BRS and its relation to atrial fibrillation recurrence. It found that RFCA significantly reduced BRS, and larger BRS reductions were associated with a lack of atrial fibrillation recurrence. While his and our study concern BRS and its relationship to atrial fibrillation, Kondo et al. [27] approach the subject differently. Our study compared BRS between PAF patients and healthy individuals, while Kondo and colleagues examined how a specific treatment (catheter ablation) affected BRS and potential atrial fibrillation recurrence. However, both studies suggest a link between changes in BRS and atrial fibrillation, whether in its presence compared to healthy individuals or in its recurrence following a specific treatment.

In our study, BEI, a measure used to quantify the effectiveness of the baroreflex, was also evaluated. PAF patients showed a lower BEI independently of the period of analysis (for further details of this analysis, see Supplementary Materials, Table S2), indicating a reduced ability of the baroreflex system to control blood pressure. This behavior might potentially contribute to the maintenance and progression of AF. However, the precise relationship between BEI and AF stills needs further evaluation in larger clinical trials. 

### 4.2. Autonomic Nervous System and Its Impact on BRS

One of the main determinants of baroreceptor function is the activity of the autonomic nervous system (ANS), which, upon postural baroreceptor unloading, elicits adjustments in sympathetic and parasympathetic outflow to the cardiovascular system, increasing both the heart rate and vasoconstriction [38,39,40,41]. Our previous study suggested the presence of ANS disturbances in cardiovascular regulation in patients with PAF, as evidenced by diminished heart rate variability during HUT [30] together with the delay in the baroreflex activation immediately after tilting up, as shown in Figure 2. These observations align with previous experiments, suggesting that the arterial component of the baroreflex remains unchanged during tilt, while the cardiopulmonary contribution decreases [42,43,44]. This novel insight may explain the sustained increase in BP in the PAF group despite the reduced BRS slope and BEI, emphasizing the complex interactions between cardiovascular regulation and ANS in PAF patients.

### 4.3. Potential Implications for PAF Patients

Our findings of simultaneously reduced BRS during the adaptation period to upright position and BEI, along the whole period of evaluation including in the supine position, represent a marker of arterial baroreflex dysfunction in patients with PAF. The discrepancy between our data and those obtained in healthy subjects to determine the BRS and BEI during HUT implies a distinct physiological response in patients with PAF. Several studies have reported a reduction in the BRS slope using spontaneous time-domain (sequence) BRS methods [38,42,43,44,45]. Also, Pitzalis and co-workers [44] found discrepancies between the results of resting spontaneous BRS and the BEI assessed at rest using the sequence method in a study conducted in healthy subjects with unexplained syncope and control subjects. This further underscores the need to understand the underlying reasons for the variability in BRS results and calls for more in-depth studies examining the specific mechanisms at play. Nevertheless, our study also allows us to conclude that the BEI is a better index of baroreflex impairment than the BRS, as its variation shows significant differences between the control subjects and the PAF patients even at rest. Therefore, we believe that further studies should be conducted to understand its role in stratifying the risk of recurrence of AF and the risk of developing complications related to AF such as stroke or heart failure, to predict the time to intervention and to develop novel treatments targeting the ANS. The ongoing study titled “Baroreflex Sensitivity in Patients Undergoing Ablation of Atrial Fibrillation (BARO-AF)” conducted by the Poitiers University Hospital in France is such an example [46]. It explores the relationship between BRS and AF recurrence and also investigates the prognostic value of BRS for AF burden after one year, in comparison to other biological and imaging parameters. Undoubtedly, this research will shed light on the role of the autonomic baroreceptor reflex function in PAF episodes. 

### 4.4. Hemodynamic Influences and Their Effects on the Modulation of the Baroreflex

A critical perspective that is often underrepresented in discussions of HUT, particularly when dealing with complicated conditions such as PAF, is the role of the modulation of preload and the resulting impact on venous return and central volume dynamics. In the context of the cardiovascular system, head tilt can be considered a strong modulator of preload. It leads to a dynamic shift of blood out of the central compartments, challenging central volumes, a phenomenon that has profound implications for the venous side of the circulation. When central volumes are challenged—either by HUT or, more intensely, by interventions such as lower body negative pressure (LBNP)—this has a consequential impact on the regulation of arterial resistance. As arterial resistance changes, we observe fluctuations in BP. These fluctuations are crucial because they can activate the baroreflex mechanism, leading to a modulation of heart rate. These findings are not limited to physiological observations. When we look at pathophysiological conditions, especially complex diseases such as atrial fibrillation, the subtleties become even clearer. The mechanisms underlying the influence of modulations of preload on venous return on baroreflex function in patients with lone PAF may be more complex, underscoring the importance of considering these variables when interpreting the data. These claims are supported by model-based work that has looked in depth at the cardiovascular system. For example, in their seminal study, Mukkamala and Cohen [46] developed a forward model-based approach to validate the identification of the cardiovascular system. Their results illustrate the intricacies of cardiovascular dynamics and how external influences can modulate systemic performance. Another remarkable study by Aletti et al. [47] highlighted a startling observation. They found that cardiac output is not a significant contributor to low-frequency mean arterial pressure variability. These findings provide an important background for the discussion of the influence of preload modulation on baroreflex sensitivity, especially in patients with lone PAF. As we incorporate these findings and expand our understanding, it becomes imperative to explore these hemodynamic subtleties more deeply. Future research could benefit from incorporating such models and investigating their direct and indirect effects on baroreflex function, particularly in conditions such as PAF.

### 4.5. Addressing Causality, Association, and Confounders

Our study design, being a case–control study, primarily identifies associations rather than causality. While we observed associations between PAF and baroreflex dysfunction, it is essential to acknowledge that these findings do not establish a direct causative relationship. To address potential confounders, we advanced statistical methods including multivariable regression analyses adjusting for age, sex, BMI, baseline BP, and HR. Despite these adjustments, the observed differences in baroreflex function between lone PAF patients and controls persisted, strengthening the validity of our findings and supporting the credibility of our conclusions (for further details of this analysis, see Supplementary Materials, Table S3).

### 4.6. Study Limitations

Our study had certain limitations. First, our sample size was relatively small and potentially lacked diversity in age, sex, and other demographic or health characteristics, which may have limited the generalizability of our findings. 

Moreover, we employed the sequence method as the sole technique to determine the baroreflex sensitivity. Although this method is widely accepted, other methodologies may yield additional or differing results. 

Lastly, our study did not include measurements beyond the 5 min of tilting up, which did not allow a comparison of baroreflex function during active standing, and prolonged HUT could not be performed. However, since the study’s purpose was to evaluate the rapid adaptations to the upright position, it is widely accepted that rapid short-term adjustments in the first two minutes of HUT are mediated by autonomic control [45].

## 5. Conclusions

The primary aim of our study was to investigate the behavior of baroreceptor reflex control in patients with lone PAF both at rest and during HUT by evaluating BRS and BEI. Our results indicate a pronounced impairment of baroreflex function in PAF patients compared to healthy subjects. While the healthy subjects consistently showed effective BRS responses to blood pressure changes, the PAF patients exhibited significant baroreflex dysfunction. Notably, the BEI provided a more descriptive measure of this dysfunction than the BRS. These observations suggest that the BEI could serve as a potential marker for predicting the recurrence of AF or assessing the efficacy of ablative interventions. Given the complicated relationship between baroreflex function and PAF episodes, further research is essential. A deeper understanding of the role of ANS in AF could pave the way for more individualized therapeutic strategies and prevention measures. 

## Figures and Tables

**Table 1:** Comparative clinical characteristics at rest for patients with paroxysmal atrial fibrillation and healthy control subjects.

Variable	PAF Group	HI Group
	(*n* = 68)	(*n* = 34)
Age, years	53 ± 14	49 ± 12
Male gender, %	47.0	36.3
BMI	26.9 ± 3.6	25.0 ± 2.7
Heart rate, bpm	70 ± 10	72 ± 5.8
Systolic blood pressure, mmHg	121 ± 4.3	115 ± 8.3
Diastolic blood pressure, mmHg	81 ± 5.5	76 ± 5.5

PAF = paroxysmal atrial fibrillation; HI = healthy individual; BMI = body mass index; bpm = beats per minute. Data expressed as mean ± SEM or %; *p* = NS.

## Data Availability

The data that support the findings of this study are available from the corresponding author upon reasonable request.

## Supplementary Materials

The following supporting information can be downloaded at: https://www.mdpi.com/article/10.3390/jcm12185857/s1, Table S1: Multiple Regression Analysis for Baroreflex Sensitivity (BRS) at TA5 Phase; Table S2: Sensitivity Analysis for Baroreflex Sensitivity (BRS); Table S3: Sensitivity Analysis for Baroreflex Effectiveness Index (BEI) with respect to the lone PAF Group.
