# Supplementary material for: Orthostatic Stress and Baroreflex Sensitivity: A Window into Autonomic Dysfunction in Lone Paroxysmal Atrial Fibrillation
Source: J Clin Med. 2023 Sep 8;12(18):5857. doi: 10.3390/jcm12185857 (PMC10532155; doi:10.3390/jcm12185857)
Supplement: Supplementary file 1 [file jcm-12-05857-s001.zip › jcm-2526838-supplementary.pdf]

Supplemental material

Bias evaluation

In human studies, the potential for bias remains a major concern. Such biases, if not addressed, can undermine the validity of the results and consequently affect the interpretation of the underlying pathophysiology based on those results. For this reason, although we applied restriction and matching methods, we undertook a rigorous assessment of potential biases that may have occurred during the recruitment, selection and implementation phases of our lone PAF study. The aim of this supplementary section is to provide readers with a transparent and detailed overview of the methods we used to detect, measure and control for these biases. For that, a multiple regression analysis to control for confounding factors was conducted as well as a sensitivity analysis to test the robustness of our results with respect to the choice of variables. Additionally, we also addressed biases specific to our study. The details of these analyses follow.

Multiple regression analysis: The impact of potential confounders on our primary outcomes BRS and BEI was addressed by a multiple regression analysis. The model included age, BMI, BP and HR as covariates. For BRS during the TA5 phase, the regression model was significant ($F(5, 96) = 15.32$, $p<0.001$), explaining approximately 44% of the variance ($R^2 = 0.44$). After adjusting for confounders, the BRS values remained significantly lower in the lone PAF group compared to the control group (beta = -3.6, $p<0.05$). For BEI during the TA5 phase, the model was also significant ($F(5, 96) = 12.21$, $p<0.001$), accounting for 40% of the variance ($R^2 = 0.40$). BEI in the lone PAF group was still significantly lower even after adjustment (beta = -12.7, $p<0.01$) (Table 1).

Table S1: Multiple Regression Analysis for Baroreflex Sensitivity (BRS) at TA5 Phase. This analysis is focused on the effects of our main predictors, which provide actionable insights into the relationship between variables such as age, BMI, the presence of lone PAF and baseline BP and HR. The omission of the intercept values is due to its limited practical interpretation in the context of our study as scenarios such as an age of zero or a HR of zero have no biological significance.

Variable	Beta Coeff.	SD	t-Value	p-Value
Intercept	-	-	-	-
Age	-0.12	0.05	-2.4	<0.05
BMI	0.05	0.03	1.67	=0.10
Baseline BP	0.21	0.07	3.0	<0.01
Baseline HR	-0.08	0.04	-2.0	<0.05
Lone PAF group	-3.6	1.2	-3.0	<0.01

Sensitivity Analysis: To test the robustness of our findings, sensitivity analyses were conducted by sequentially excluding each potential confounder from the multiple regression models. The significance and direction of the effects remained consistent across all models for both BRS and BEI, confirming that our results are robust to the inclusion or exclusion of these variables. Specifically, the effect sizes for the difference in BRS and BEI between the lone PAF and control groups ranged from a beta coefficient of -3.4 to -3.8 for BRS (Table 2) and a beta coefficient of -12.1 to -13.2 for BEI (Table 3), across all sensitivity models. None of these changes altered the statistical significance of the primary outcomes.

Table S2: Sensitivity Analysis for Baroreflex Sensitivity (BRS). Since the beta for the lone PAF group remains consistent across all these models, this highlights the robustness of BRS results for the lone PAF group, regardless of the inclusion or exclusion of other variables.

Excluded variable	Beta for lone PAF Group	p-Value
None	-3.6	<0.05
Age	-3.4	<0.05
BMI	-3.5	<0.05
Baseline BP	-3.7	<0.05
Baseline HR	-3.8	<0.05

Table S3: Sensitivity Analysis for Baroreflex Effectiveness Index (BEI) with respect to the lone PAF Group. The consistency of the beta values in the different models underlines the reliability of our

BEI results for the lone PAF group, regardless of whether other variables are included or omitted.

Excluded variable	Beta for lone PAF Group	p-Value
None	-12.7	<0.01
Age	-12.1	<0.01
BMI	-12.5	<0.01
Baseline BP	-13.0	<0.01
Baseline HR	-13.2	<0.01

Specific biases

(i) Sample bias: The study was conducted in a single hospital, which may limit the generalisability of our results. However, the patient and control groups were carefully matched for age, sex and other demographic factors, which justifies the internal validity of our study.

(ii) Volunteer bias: The study relied on volunteer participants, which could introduce bias. However, as both the control group and the group of lone PAF patients responded to HUT in a manner consistent with known physiological responses, the likelihood of volunteer bias significantly affecting our conclusions appears to be low.

(iii) *Exclusion criteria*: While our exclusion criteria were stringent, they were essential to isolate the effect of PAF alone on baroreflex function. Given that our results showed significant differences in BRS and BEI between lone PAF patients and controls, the exclusion criteria seem justified.

(iv) Measurement and instrumentation: All measurements were carried out with a calibrated device, the Taskforce Monitor (CNSystem, Graz, Austria), with pre-processing steps to minimise signal interference. While this raises the possibility of measurement bias, the consistency of observed changes in BRS and BEI in lone PAF patients compared to control subjects suggests that such bias would be systematic and, therefore, unlikely to affect the internal validity of the study.

(v) Unmeasured confounders: Although many potential confounders were controlled by our study design, there may still be unmeasured variables that influence baroreflex function, such as stress or circadian rhythms. However, given the significant differences between the

lone PAF and control groups in BRS and BEI, the influence of such unmeasured confounders is likely to be minimal.
